# Disinvesting in the future leadership of global health has already begun: What can we do about it?

**DOI:** 10.1371/journal.pgph.0005310

**Published:** 2025-10-29

**Authors:** Shashika Bandara, Jane K. Fieldhouse, Inosha Alwis, Lucía Abascal Miguel, Canice Christian, Nelson Evaborhene

**Affiliations:** 1 Department of Global and Public Health, School of Population and Global Health, McGill University, Montreal, Québec, Canada; 2 Grand Challenges, University of California Davis, Davis, California, United States of America; 3 Australian Centre for Health Services Innovation (AusHSI) and Centre for Healthcare Transformation, School of Public Health & Social Work, Queensland University of Technology, Brisbane, Queensland, Australia; 4 Department of Community Medicine, Faculty of Medicine, University of Peradeniya, Peradeniya, Sri Lanka; 5 Institute for Global Health Sciences, University of California, San Francisco, San Francisco, California, United States of America; 6 Department of Epidemiology and Biostatistics, University of California, San Francisco, San Francisco, California, United States of America; 7 Faculty of Health Science, University of the Witwatersrand, Johannesburg, South Africa; University of Minnesota, UNITED STATES OF AMERICA

## Abstract

Following the abrupt and significant funding cuts by the U.S. and increasing retreat by high-income countries from development aid for health, global health as a field requires reimagining and urgent solution building by all parties involved. In this essay, we aim to draw attention to an important and urgent challenge that deeply affects our collective future: *the destruction of global health training opportunities and the weakening of future global health leadership*. If we do not approach this challenge with a sense of urgency, global health research and training face irreversible shifts, weakening global preparedness to face future pandemics, address climate crisis, and achieve global goals such as universal health coverage or health for all. We outline existing best practices that we can build on and pathways to build better approaches in global health training.

## Introduction

“We are living through the greatest disruption to global health financing in memory,” Dr. Tedros, the Director General of the World Health Organization (WHO) said at a press conference describing the impact of recent policy changes by the United States (U.S.) [1]. Isolationist policies enacted by the U.S. have upended global health funding, severely affecting global health delivery, infrastructure and governance, and significantly damaging opportunities for trainees across the world [2,3]. Many high-income countries (HICs) have also cut the development aid for health budget compounding this impact [4].

Between 2021 and 2023, the U.S. was the single largest donor in development assistance of health in low- and middle-income countries (LMICs), contributing to over 30% of all health assistance ($8.3 billion) [5]. An estimated 25 million deaths are projected in the next 15 years due to funding cuts on major global health programs [6]. As noted, the consequences of the sudden funding freeze by the U.S., reduction of aid for health by high-income countries, coupled with isolationist policies are well documented. Experts warn of the lasting effects on health programs, national budgets, and the well-being of populations around the globe [1,7].

In this essay, however, we aim to draw attention to an equally important and urgent challenge which deeply affects our collective future: *the destruction of global health training opportunities and the weakening of future global health leadership*. If we do not approach this challenge with a sense of urgency, global health research and training face irreversible changes, weakening global preparedness to face future pandemics, address climate crisis, and achieve global goals such as universal health coverage or health for all.

To understand the scale of the problem, we examine the impact of the Trump administration’s targeted attacks on global health training and research in the U.S. and worldwide. Public health institutions such as the National Institutes of Health (NIH) and the U.S. Centers for Disease Control and Prevention (CDC) are facing existential threats with cascading effects on a wide network of local and global institutions, many of them interconnected and dependent on their support and collaboration [8,9]. NIH, the largest funder of biomedical research in the world, including research on diseases with the highest global burden such as tuberculosis and HIV, has faced major disruptions; as of September 2025, nearly $2.4 billion in grant funding was lost, affecting more than 2,400 grants [10]. International NIH collaborations have been halted, while the withdrawal rate of NIH grant applications has doubled, effectively closing doors for both U.S. based and international health researchers [8,9]. Importantly, the situation remains in flux, creating a state of heightened uncertainty. For example, a recent court ruling temporarily reversed a portion of the cuts, the government has appealed the decision, leaving the future of NIH and aid funding highly uncertain [11].

This gutting of research funding has triggered a domino effect across the global public health and biomedical research ecosystems. NIH’s deep integration with universities around the world means that institutions outside the U.S., both in the Global North and South, are also affected [12]. For example, in the Global North, Australian scientists are being surveyed to disclose their institution’s compatibility with the United States’ foreign and domestic policy, with possible terminations following investigation. The president of the *Australian Academy of Science* has written to the Australian government urging to safeguard country’s research strategies, since at minimum 386 million of its research funds come from the U.S. [13]. Canadian researchers tell a similar story with NIH grants being canceled. Researchers from Canada highlight the current feeling of devastation and uncertainty as “funeral for our careers,” [14].

In the Global South, most research partnerships remain under threat. For example, up to 70% of South Africa’s medical research, including HIV/AIDS research, is supported by the NIH [15]. More broadly, in 2023, NIH had collaborations with nearly 1200 research organizations in Asia, 1200 in Africa, and 500 in Latin America [16]. The training and capacity building opportunities of young public and global health professionals in these institutions are now largely jeopardized [16].

Critically, these setbacks come just as African-led institutions were beginning to gain international recognition and autonomy. Centers such as the University of Global Health Equity in Rwanda (UGHE) [17], the African Centre of Excellence for Genomics of Infectious Diseases in Nigeria [18], and the South African Medical Research Council [19] have emerged as powerful nodes in the global health ecosystem. The Africa CDC’s Kofi Annan Global Health Leadership Program—now training its fifth cohort—illustrates how the continent has been actively investing in its own leadership pipeline, with over 80 fellows trained or in training across AU member states [20]. Universities like Makerere, Wits, and UGHE continue to cultivate equity-centered models of public health education that respond to regional realities. These achievements mark a critical inflection point in shifting global health leadership southward. However, their growth depends on sustained collaboration, mobility, and fair access to global resources—now all under threat due to funding freezes and policy changes in donor nations.

This funding crisis, if not addressed swiftly, halts the possibility of advancing collaborative research and training on pandemic preparedness, climate crisis preparedness, disease surveillance and monitoring, mitigating the impact of HIV/AIDS or TB, among others – dramatically decreasing opportunities for young trainees. This crisis also exacerbates the inequities within global health training by drastically reducing scholarships and research support for early-career researchers from LMICs, reinforcing the already skewed Global North-centric model of global health [21,22]. In short, we will be poorly prepared for global health crises while our training opportunities in global health will heavily perpetuate harmful models of elite capture [23,24].

Adding to this, the Trump administration has actively undermined research on equity, gender, structural discrimination, and the social determinants of health. These changes weaken the U.S.’s capacity to tackle its own health inequities and damage global efforts toward health equity, especially in the context of Sustainable Development Goal 3, which places equity at the heart of health for all.

The criminalization and stigmatization of international students, backed by physical and structural violence, pose further structural barriers to advance the development and training of global health leaders [21]. Harvard University, facing a first of its kind threat, had their ability to have international students enrolled in their programs blocked [25]. Universities such as Johns Hopkins, known for their expertise in global health, have fewer opportunities for international student graduates with diminishing grant funding [8]. In addition to loss of opportunities, there is also a culture of fear among international trainees in the U.S., caused by the unsafe environment created by the U.S. government. Our efforts to strengthen bi-directional knowledge flow, already threatened by challenges such as visa and passport inequities and the unjust implementation of immigration laws, is further threatened by the stigmatization and exclusion of international scholars [21].

Against this backdrop, as a group of global health trainees with ties to the U.S., Mexico, Sri Lanka, Nigeria, Canada, Australia, and South Africa, we recognize the urgency of confronting this crisis head-on. Therefore, we offer solution pathways as a starting point for collective action to protect the future of global health leadership.

## Pathways for sustaining training of future leaders in global health

Driven by necessity, we call for urgent action to mitigate the substantial damage caused by the policies outlined above to global health education and training.

Multi-level and multi-party solutions are required to address the diminishing training opportunities, the resulting impact on the wellbeing of trainees, and to stop the exodus of future leaders from the global health sector. In terms of levels, individual, community, and institutional (including governance) level solutions are required. The responsible and leading parties can range from principal investigators (PIs), educational institutions, funding bodies, and government ministries and or departments. While some solutions need to be codified in policy and law, we also require solutions that shift our culture in global health. Specifically, we consider this an apt moment to change our collective mindset and structural foundations which has assigned the Global North as the default setting for excellence in training in global health [22,24]. We recognize the efforts by countries in the Global South to move away from Global North funding dependency within global health and call for structural changes that allow for situating more global health training opportunities in the Global South [26].

*Individual- and community-level efforts* are already being initiated by many PIs and the global health community by sharing opportunities with impacted trainees and by offering spaces of care. Many of these efforts, however, are initiated at personal discretion. These personal initiatives can often lose steam, especially since PIs also navigate challenges due to funding cuts and policy directives damaging global health [27]. Thus, we advocate for more structured and intentional community building, information sharing, and building spaces for honest conversations. For example, the McGill Reimagining Global Health course, offered via McGill Global Health Summer Institutes, offers a space for evidence-informed, honest conversations that lead to opportunities for community building in global health [28]. Other examples include PIs actively providing alternative opportunities to affected trainees to maintain research training and standing by the trainees by mitigating structural barriers. These barriers could be visa challenges, challenges to trainee status, or blocked access to university resources (e.g., library access) among others.

*At the institutional level*, this is the time for academic and research bodies to double-down on principles of equity, diversity, and inclusion. It is highly necessary to “care for those who care for your communities” by strengthening support systems and recognizing shortcomings within institutions to support students [29]. Leaders within the institutions at the highest level (e.g., principals, chancellors, and deans) as well as those at intermediate levels (e.g., department heads, professors, lab leads) should intentionally commit to mitigating structural barriers arising from the shifting status quo in global health. Harvard University has led by example where they continue to stand by their trainees via sustaining training opportunities while navigating threats from the U.S. government targeting international students and others [25]. The University of Toronto has now joined as an ally by creating a pact allowing to host affected international Harvard students [30].

Given the legal and structural barriers within the U.S., this could also be an opportunity for the global health community to strengthen Global South centers of education and base educational programs in partnership with Global South collaborators. Starting points for these opportunities already exist. Examples include Duke Global Health Institute’s priority partnership locations, leadership by Global South universities including National University of Singapore, and UGHE, Rwanda. Rather than viewing the shift of educational programs to a Global South-centered model as a stop gap solution, this could be an opportunity to genuinely reimagine global health education, with regional experts leading research on topics relevant to those regions with global collaboration. This approach aligns with calls for strengthening regional leadership and resource building following inequitable responses to the COVID-19 pandemic and mpox outbreaks where HICs prioritized themselves [24,31,32].

As we envision and implement solutions to address the crisis of diminishing training opportunities and the erosion of effective future global health leadership, it is essential to actively discourage elite capture of opportunities and solutions [33]. The privileged few dominating spaces in global health continues to be a challenge supported by current ‘soft money’ funding models and lack of intentionality to shift leadership to the Global South where relevant [24]. This is a time for funders to interrogate the structures they have built, their neo-colonial foundations, and consider the question “how can we sustain global health training to build future generations of leaders with *equity* at the center?”

Finally, we want to remind readers that the core of these challenges lies within political decision making – both domestic and global. Therefore, as we build solutions at the levels outlined above, using existing examples and platforms, it is highly necessary to advocate for better policy solutions implemented by national and sub-national governance leaders. One example is the Bethesda Declaration, signed by leaders within the scientific research community in the U.S. and leading research organizations, who stood together with NIH staff to challenge harmful changes to the NIH [34]. Reversing the current trend of gutting global health funding, re-directing funding resources to defense, and combating the isolationist policies that kill knowledge creation and sharing within global health will require concerted effort from all. Therefore, we ask decision-makers within our governments to commit to political leadership for a better future, including funding in global health. We also ask our communities to draw attention to harmful policies and advocate for better policies, not allowing governments or institutions to weaken global health in silence. We collate all our suggested approaches in Table 1.

**Table 1:** Suggested solutions for sustainable training of global health leaders.

Level of solution building	Suggested approaches
Personal and community	Share alternative opportunities for affected trainees to consider Care for the wellbeing of affected trainees Build team culture that prioritizes support and wellbeing of trainees Be considerate of the limitations and pressures PIs are facing Build spaces (e.g., courses, forums) to critically examine global health and discuss alternative pathways Highlight poor policies affecting global health and advocate for anti-isolationist and health-justice-centered policies
Academic institutions	Build spaces (e.g., courses, forums) to critically examine global health and discuss alternative pathways Build contingency measures for affected students via alternative funding, legal action, and partnerships Commit to shifting and centering global health training in the Global South – build on existing platforms Strengthen equity, diversity and inclusion efforts within institutions via funding and administrative commitment Support intra-African/South-South mobility and co-led research programs
Funders	Commit to shifting the funding architecture to reduce dependency on the Global North institutions Analyze own funding policies to reduce neo-colonial funding policies and structures Commit to restructuring funding to reduce reliance on Global North institutions Support South-led consortia and capacity-building platforms like Africa CDC Reassess grant models that perpetuate inequity or elite capture
Governmental decision-makers	Push back against isolationist policies affecting global health funding Advocate for enacting strong national and regional policies to counter discrimination and hate against diverse and international students Build collaborative partnerships with governments and philanthropic organizations to address the current funding crisis Create regional policies to initiate and sustain trainee programs to build recognized leadership in global health Support immigration frameworks that safeguard international scholars Increase domestic health research and development investment and partner with regional funders

As many have argued recently, and in the past, a crisis is an opportunity to interrogate the weaknesses of our structures and produce meaningful solutions. The current global health funding and political crisis is a moment for us to shift our thinking and our structures toward equity and to strengthen Global South leadership in global health. A central tenet of this commitment is to strengthening future global health leadership by sustaining opportunities for trainees worldwide with equity at the center.
